# Primary mediastinal ependymoma: A case report and literature review

**DOI:** 10.1097/MD.0000000000017686

**Published:** 2019-11-01

**Authors:** Wei-biao Ye, Jian-ping Zhou, Yong-qiang Xu, Bi-yan Lu, Zhong-jun Li

**Affiliations:** a Department of Thoracic Surgery and Pathology, Dongguan People's Hospital of Southern Medical University; b Department of Basic Medical Sciences, Dongguan Polytechnic, Dongguan, Guangdong Province, China.

**Keywords:** ependymoma, immunohistochemistry, mediastinal neoplasm, metastasis

## Abstract

**Rationale:**

Ependymomas are neuroepithelial tumors that typically occur in the central nervous system. Ependymomas arising in the mediastinum are exceedingly rare, with only approximately 9 isolated cases reported in the literature to date.

**Patient concerns:**

A 35-year-old woman was referred to our hospital with complaints of progressive back pain for 3 months. Physical examination revealed decreased breathing sounds and tenderness. Contrast-enhanced computed tomography showed a soft tissue mass with heterogeneous enhancement in the right posterior mediastinum.

**Diagnoses:**

The diagnosis of primary mediastinal ependymomas (PMEs) was confirmed by postoperative histopathologic examination.

**Interventions and outcomes:**

The patient underwent surgical resection of the tumor and experienced local recurrence with neck metastasis 2 years postoperatively. She underwent reoperation for the recurrent tumors and received postoperative radiotherapy and adjuvant chemotherapy. Two years later, the patient is doing well, with no evidence of tumor progression or recurrence.

**Lessons:**

Since PMEs are exceedingly rare, treatment options are limited. Surgical resection seems to be the mainstay of treatment. Further evidence-based studies are required to prove the benefit of radiotherapy and chemotherapy in the treatment of PMEs.

## 1 Introduction

Ependymomas represent a relatively rare group of glial tumors that originate from the ependymal lining of the ventricular system and central canal of the spinal cord.^[1]^ Extracranial or extraspinal ependymomas are quite unusual, and their occurrence in the mediastinum is extremely rare.^[2–8]^ According to clinical characteristics, all patients with primary mediastinal ependymoma (PME) are adult women. Although occasional cases with regional lymph node or distant metastasis have been reported, PMEs are considered as low-grade tumors and have a relatively favorable prognosis.^[9]^ Due to their relative rarity, optimal treatment strategies are limited and not well-established. Surgical resection with a tumor-free margin seems to be the mainstay of treatment. The benefit of radiotherapy and adjuvant chemotherapy in the treatment of PMEs remain to be proven by conducting further evidence-based studies.

## 2 Case report

**Figure 1:**
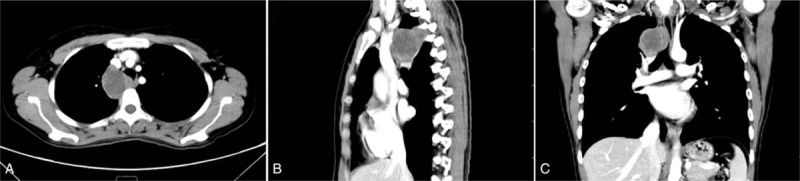
Contrast-enhanced computed tomography of the chest reveals a well-defined mass in the right posterior mediastinum: axial (A), sagittal (B), and coronal (C) reconstructions.

**Figure 2:**
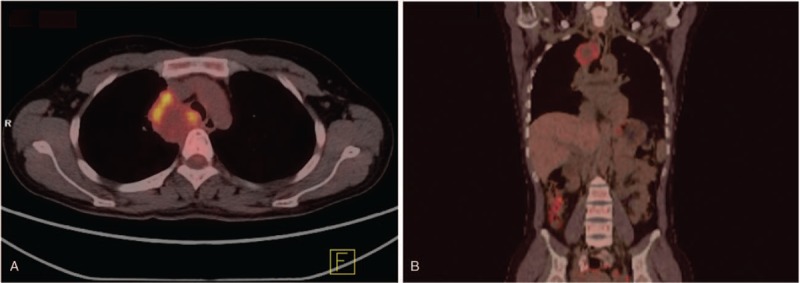
Positron emission tomography-computed tomography shows fluorodeoxyglucose uptake in the mediastinal tumor (maximum standardized uptake value 3.45).

A 35-year-old, nonsmoking, Chinese woman was admitted to our hospital with a 3-month history of progressive back pain. She denied any injury or significant medical history. Physical examination revealed decreased breathing sounds and tenderness in her right chest. Cardiac examination was normal, with a blood pressure of 120/65 mm Hg and a heart rate of 82 beats per minute. Routine laboratory test results were unremarkable. Chest roentgenogram showed a right paratracheal opacity. Computed tomography (CT) identified a well-defined soft tissue mass measuring 6.0 cm × 5.5 cm × 3.5 cm in the right posterior mediastinum at the T2-T3 level (Fig. 1). On contrast enhanced images, the lesion exhibited mild heterogeneous enhancement. Fluorine-18 fluorodeoxyglucose positron emission tomography (FDG-PET) showed that the tumor was metabolically active, with a maximum standardized uptake value of 3.45 (Fig. 2). There was no abnormal FDG uptake in other areas.

**Figure 3:**
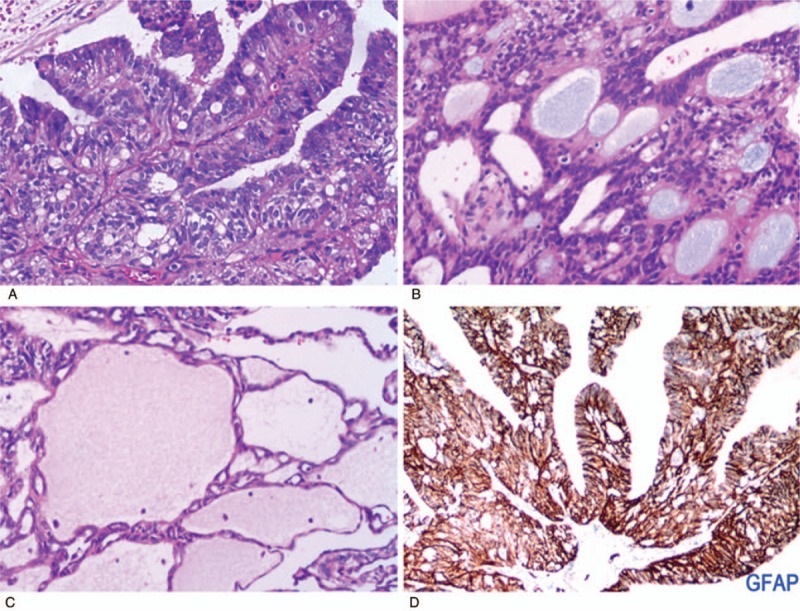
(A–C) Histologic appearance of the tumor showing cuboidal or columnar cells arranged in a papillary, trabecular, or cystic architecture (hematoxylin and eosin). (D) Immunohistochemical staining of the tumor showing positivity for GFAP.

The patient underwent gross total resection of the tumor. The operative findings revealed no evidence of invasion in the thoracic vertebra or other mediastinal organs. Overall, the resected tumor was predominantly solid with focal cystic degeneration. Microscopically, the tumor was composed of cuboidal or columnar cells arranged in a papillary, trabecular, or cystic architecture (Fig. 3). Characteristic perivascular pseudorosettes were frequently observed. Mitoses were observed only occasionally (1/10 high-power fields [HPFs]), and necrosis was absent. Immunohistochemical examination showed that the tumor cells were positive for Vimentin, GFAP, CD56, ER, and PR. Based on these findings, the tumor was diagnosed as PME (WHO grade II).

The patient declined any further treatment and was followed-up in the outpatient clinic. Unfortunately, local recurrence with neck metastasis was detected through enhanced CT 2 years after the initial surgery. The patient underwent reoperation for the recurrent tumors and received postoperative radiotherapy (59.4 Gy in 1.8 Gy fractions) with concomitant temozolomide (75 mg/m^2^/d), followed by 10 cycles of adjuvant temozolomide (150 mg/m^2^/d for 5 days every 4 weeks). Two years later, she remains well with no evidence of tumor progression or recurrence.

## 3 Discussion

Ependymomas are neuroepithelial tumors with ependymal differentiation that typically occur in the central nervous system.^[1]^ They constitute approximately 1.9% of all primary central nervous system tumors in adults.^[10]^ Reported extraneural sites for ependymomas include the ovary, broad ligament, sacrococcygeal region, lung, and mediastinum. PMEs are extremely rare, with only 10 cases reported in the literature to date, including the present case (Table 1).^[2–8]^ Until now, all reported cases of PMEs were sporadic. With respect to clinical characteristics, all patients with PME were women aged 35–71 years (median age: 44 years). These patients had no clinical symptom or had only nonspecific symptoms, such as back pain and dyspnea. In addition, laboratory and radiologic findings were nonspecific. However, PMEs usually appear as a well-defined mass with variable homogeneous to heterogeneous enhancement on CT or magnetic resonance imaging. The scanning acquisitions may vary depending on the presence of underlying cystic degeneration, hemorrhage, and calcification.

**Table 1:**
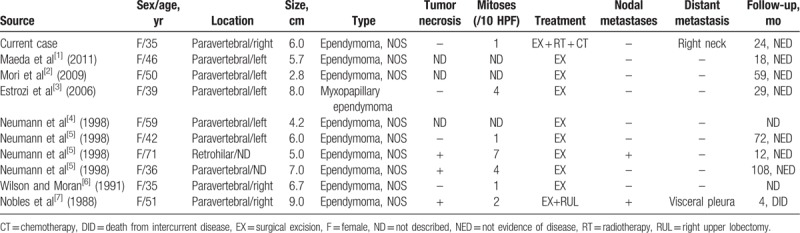
Overview of the published cases of primary mediastinal ependymoma.

Macroscopically, PMEs are described as fairly well-circumscribed soft masses measuring 2.8 to 9.0 cm in diameter. On the cut section, the tumors show mainly solid and partially cystic components. Histopathologically, these tumors demonstrate a variety of architectural patterns, including cribriform, trabecular, cystic, papillary, and solid formations. Characteristic perivascular pseudorosettes and true rosettes are frequently present. Tumor cells are cuboidal to columnar, containing abundant clear to granular eosinophilic cytoplasm, with round to oval nuclei. The mitotic rate ranges from 1 to 10 per 10 HPF. Necrosis is observed in some cases. Immunohistochemically, the tumor cells are positive for GFAP, CK7, ER, and PR, but negative for S-100, TTF-1, CAM5.2, and CK20. In particular, the CD99 expression pattern in PMEs is usually weak and patchy compared with the strong and diffuse staining observed in central nervous system cases.^[2]^

Primary posterior mediastinal masses are relatively uncommon. The majority of primary neoplasms in this location are of neurogenic origin.^[11]^ In adults, the most frequent subtypes are schwannomas and neurofibromas, while ganglioneuroblastoma and neuroblastoma occur most commonly in infants and children. Other less common but important differential diagnostic considerations include lymphoma, sarcoma, extra-gastrointestinal stromal tumor, intrathoracic desmoid tumor, and granular cell tumor.^[12–14]^ In addition, noncancerous diseases such as foregut cysts, Wegener granulomatosis, and thoracic extramedullary hematopoiesis may occur in the posterior mediastinum.^[15]^

There is no plausible unifying concept for the pathogenesis of PMEs. Germ cell origin, displaced ependymal rests, and neometaplasia are the most well-known theories. Since these tumors predominantly occur in women in the ovary, broad ligament, and mediastinum, they support the theory of germ cell origin.^[2,16]^ However, lack of conventional teratomatous elements or other germ cell components seems to rule out this possibility. On the contrary, the paravertebral and posterior mediastinal location together with a microscopic view of adjacent sympathetic ganglia and nerves supports the displaced ependymal rests theory.^[6,17]^

In summary, all 10 patients with PME exhibited an indolent clinical course, and 8 were followed-up. Among them, 6 patients were asymptomatic and showed no evidence of recurrence (follow-up time 12–108 months). Of the remaining 2 patients, 1 died due to complications 4 months postoperatively, and the other reported recurrence with distant metastases. Taken together, PMEs are slow-growing, low-grade tumors. Surgical resection with a tumor-free margin is the primary treatment of choice. There are no prior reports of chemotherapy or radiotherapy for PME. We have described the 1st case that received chemotherapy and radiotherapy in addition to surgical resection.

## 4 Conclusion

The PMEs are exceedingly rare with only a limited number of published reports worldwide. The differential diagnosis is challenging due to the rarity of the disease and its nonspecific clinical presentation. Complete surgical excision remains the primary treatment of choice. Further evidence-based studies are required to establish the benefit of chemotherapy or radiotherapy in the treatment of PMEs.

## Acknowledgments

The authors thank Yuan-qiu Liang for assistance with pathology images and immunohistochemical staining. In addition, they thank Wei-tao Li for helpful suggestions and technical assistance.

## Author contributions

**Resources:** Jian-ping Zhou.

**Supervision:** Zhongjun Li.

**Writing – original draft:** Wei-biao Ye, Yong-qiang Xu.

**Writing – review & editing:** Bi-yan Lu.
